# Maternal inflammatory biomarkers and neonatal characteristics as predictors of bronchopulmonary dysplasia in preterm infants: a retrospective cohort study

**DOI:** 10.3389/fmed.2025.1735582

**Published:** 2026-01-08

**Authors:** Yuanling Liu, Canran Fang, Shuai Zhao

**Affiliations:** Department of Neonatology, The Affiliated Hospital, Southwest Medical University, Luzhou, Sichuan, China

**Keywords:** bronchopulmonary dysplasia, chorioamnionitis, nomogram, platelet-to-white blood cell ratio, preterm infant

## Abstract

**Background:**

Maternal peripartum inflammation may contribute to bronchopulmonary dysplasia (BPD) in preterm infants, yet readily available hematologic predictors are underexplored. We evaluated whether the maternal platelet-to-white blood cell ratio (PWR) within 24 h before delivery, together with neonatal clinical variables, predicts BPD.

**Methods:**

We conducted a retrospective cohort study at a tertiary NICU in Southwest China, including all eligible mother–infant pairs with preterm birth (<37 weeks) between June 2020 and June 2024. BPD was defined per the 2018 NICHD criteria. Maternal demographics, obstetric complications, hematology (WBC, PLT, PWR, NLR, and PLR), and neonatal characteristics (gestational age, birth weight, sex, Apgar score, SGA, sepsis, and respiratory support) were abstracted from electronic records. Variables with *p* < 0.10 in univariate analysis were entered into multivariable logistic regression. Model performance was assessed by AUC, Hosmer–Lemeshow test, bootstrap calibration, and a nomogram was constructed.

**Results:**

Among 345 pairs, 117 infants (33.9%) developed BPD. Mothers of BPD infants had higher WBC and lower PLT, yielding a lower PWR (median 18.9 vs. 23.1, *p* < 0.001). In multivariable analysis, lower maternal PWR independently predicted BPD (aOR = 0.94 per unit increase; 95% CI, 0.91–0.98; *p* = 0.002). Additional independent predictors included chorioamnionitis (aOR = 1.94; 1.01–3.71; *p* = 0.046), male sex (aOR = 1.61; 1.00–2.59; *p* = 0.049), early-onset sepsis (aOR = 2.45; 1.04–5.77; *p* = 0.040), late-onset sepsis (aOR = 2.86; 1.47–5.56; *p* = 0.002), and mechanical ventilation >7 days (aOR = 6.74; 3.34–13.62; *p* < 0.001). Gestational age and birth weight were protective (per week: aOR = 0.68; 0.58–0.79; *p* < 0.001; per 100 g: aOR = 0.86; 0.78–0.94; *p* = 0.001). The final model showed strong discrimination (AUC = 0.866; 95% CI, 0.827–0.905) and good calibration (Hosmer–Lemeshow *χ*^2^ = 10.833, *p* = 0.211; bootstrap slope = 0.98). At the optimal probability threshold (0.763), sensitivity was 77.2% and specificity was 80.9%. A nomogram enables individualized risk estimation.

**Conclusion:**

A lower maternal PWR measured immediately before delivery, alongside established neonatal risk factors, independently predicts BPD in preterm infants. This simple hematologic marker, integrated into a nomogram, may facilitate early identification and targeted prevention strategies in high-risk dyads.

## Introduction

Bronchopulmonary dysplasia (BPD) is one of the most common and severe chronic respiratory disorders among preterm infants, particularly those born at extremely low gestational ages or birth weights (1). Despite remarkable advances in perinatal and neonatal care—including antenatal corticosteroids, surfactant therapy, and non-invasive ventilation—the incidence of BPD has remained largely unchanged over the past two decades (2). This persistence reflects a shift from the “old” fibroproliferative form to a “new” BPD characterized by arrested alveolar and vascular development (3). The condition continues to impose a substantial burden on both affected families and healthcare systems, contributing to prolonged hospitalization, recurrent rehospitalization, and sustained respiratory morbidity (4). Moreover, BPD’s consequences extend beyond infancy, predisposing survivors to chronic lung disease, pulmonary hypertension, and neurodevelopmental impairments during later life (5).

Traditionally, BPD has been defined by oxygen dependence at 28 days of life or at 36 weeks’ postmenstrual age, as per the National Institute of Child Health and Human Development (NICHD) consensus (6). While this definition facilitates standardized classification, it remains descriptive and retrospective, offering little value for early prediction or prevention. Most existing predictive models rely on neonatal demographic and clinical variables—such as gestational age, birth weight, Apgar score, and respiratory support requirements (7, 8). However, these parameters primarily reflect postnatal disease expression rather than prenatal susceptibility. Consequently, early identification of high-risk infants before or immediately after birth remains an unmet clinical need.

A growing body of evidence indicates that intrauterine inflammation and maternal immune dysregulation play pivotal roles in the pathogenesis of BPD (9, 10). Exposure to inflammatory stimuli *in utero*, such as chorioamnionitis or elevated maternal cytokines, can induce a fetal inflammatory response that disrupts pulmonary angiogenesis and alveolarization, predisposing the preterm lung to injury and impaired repair (11). Yet, while several molecular and placental markers of inflammation have been explored, their measurement is complex, time-sensitive, and impractical for routine obstetric screening. Therefore, identifying simple, cost-effective, and reliable maternal biomarkers that reflect peripartum inflammatory status may provide an attractive alternative for early risk stratification.

In recent years, hematologic indices derived from complete blood counts—such as the neutrophil-to-lymphocyte ratio (NLR), platelet-to-lymphocyte ratio (PLR), and platelet-to-white blood cell ratio (PWR)—have emerged as accessible markers of systemic inflammation across various obstetric and neonatal conditions, including preeclampsia, preterm labor, and neonatal sepsis (12, 13). Among these, PWR has shown potential as a sensitive indicator of inflammatory imbalance, reflecting platelet consumption and leukocyte activation (14). However, its role in predicting BPD risk has not been systematically investigated. Clarifying whether maternal PWR or related ratios measured immediately before delivery are associated with neonatal BPD could provide new insights into the maternal–fetal inflammatory continuum and inform early preventive strategies.

Therefore, the present study aimed to evaluate whether the maternal platelet-to-white blood cell ratio (PWR) obtained within 24 h before delivery independently predicts the subsequent development of BPD in preterm infants. In addition, other hematologic ratios, such as the neutrophil-to-lymphocyte ratio (NLR) and platelet-to-lymphocyte ratio (PLR), were explored as secondary indicators to contextualize the maternal inflammatory profile. We hypothesized that a lower maternal PWR, indicative of heightened systemic inflammation, would be independently associated with increased BPD risk after adjustment for established neonatal predictors. Furthermore, by integrating maternal hematologic indices with key neonatal clinical variables, we sought to construct a multivariable prediction model and visualize it as a nomogram for individualized risk estimation. This approach offers a pragmatic and biologically integrated framework for early identification of infants at high risk for BPD, emphasizing the maternal contribution to neonatal respiratory outcomes.

## Methods

### Study design and setting

This retrospective cohort study was conducted at the Department of Neonatology of the Affiliated Hospital of Southwest Medical University (Luzhou, Sichuan, China), a tertiary regional referral center for high-risk pregnancies and premature infants. Medical records of preterm infants and their mothers were reviewed between June 2020 and June 2024. The study protocol was approved by the institutional ethics committee (approval no. KY2025448). Given the retrospective nature of the study and use of anonymized data, the requirement for written informed consent was waived.

### Participants

All available mother–infant pairs were retrospectively identified from the hospital electronic medical record system during the study period (June 2020 to June 2024). Eligible cases were those in which the infant was born preterm (gestational age <37 weeks) and admitted to the neonatal intensive care unit (NICU) within 24 h after birth. Inclusion criteria required the presence of complete maternal clinical information and hematologic results obtained within 24 h before delivery, along with complete neonatal demographic and clinical data, including records of respiratory support and infection status. Infants were further required to have survived beyond 28 days after birth to allow a reliable assessment of bronchopulmonary dysplasia (BPD). Cases were excluded if major congenital malformations or chromosomal abnormalities were documented; if severe intrapartum asphyxia, defined as umbilical cord pH < 7.0 or a 5-min Apgar score ≤3, or early neonatal death (<7 days) occurred; or if maternal or neonatal laboratory data were incomplete. After application of these criteria, a total of 345 mother–infant pairs were included in the final analysis, comprising 117 infants diagnosed with BPD and 228 without BPD, as determined retrospectively according to standardized diagnostic criteria described below.

### Variables and definitions

The primary outcome of this study was the occurrence of bronchopulmonary dysplasia (BPD), defined according to the 2018 National Institute of Child Health and Human Development (NICHD) consensus as oxygen dependence at 36 weeks’ postmenstrual age or discharge home on supplemental oxygen. The predictor variables included both maternal and neonatal characteristics. Maternal factors comprised age, gravidity, obstetric complications such as preeclampsia, gestational diabetes mellitus (GDM), premature rupture of membranes (PROM), and chorioamnionitis, and delivery mode and antenatal corticosteroid exposure. Maternal hematologic indices obtained within 24 h before delivery included the white blood cell (WBC) count, platelet (PLT) count, and several derived inflammatory ratios. Among these, the platelet-to-white blood cell ratio (PWR)—calculated as PLT divided by WBC (both expressed in ×10^9^/L)—was the primary hematologic variable of interest, while the neutrophil-to-lymphocyte ratio (NLR) and platelet-to-lymphocyte ratio (PLR) were evaluated as secondary inflammatory indicators. Neonatal variables included gestational age, birth weight, sex, Apgar scores, small-for-gestational-age (SGA) status, early-onset sepsis (EOS), late-onset sepsis (LOS), and the requirement for mechanical ventilation exceeding 7 days.

### Data sources and measurement

Maternal demographic, obstetric, and laboratory data were extracted from the hospital electronic medical record system and verified by two independent investigators. Hematologic indices were measured in the hospital’s central laboratory using an automated hematology analyzer (Sysmex XN-9000, Japan) within 2 h of sample collection. Neonatal data—including gestational age (based on first-trimester ultrasound or last menstrual period), birth weight, Apgar scores, respiratory management, and infection outcomes—were obtained from NICU medical charts and validated by cross-checking nursing and physician records. All measurements followed the same laboratory and clinical protocols for all participants, ensuring intergroup comparability.

### Bias control

To minimize selection bias, all eligible preterm infants within the defined period were consecutively included. Data extraction and verification were independently performed by two blinded researchers, with discrepancies resolved through consensus. Measurement bias was reduced by using standardized instruments and uniform laboratory methods. Confounding bias was addressed through multivariable logistic regression, in which variables with *p* < 0.10 in univariate analysis were entered into the final model.

### Study size

The study included all available eligible cases during the 4-year period. A post-hoc sample-size adequacy check confirmed that 345 participants (117 BPD events) provided more than 90% power to detect an odds ratio ≥1.8 for key predictors (*α* = 0.05), meeting recommended events-per-variable (EPV ≥ 10) requirements for logistic modeling.

### Quantitative variables

Continuous variables were assessed for normality using the Shapiro–Wilk test. Normally distributed data were expressed as mean ± standard deviation (SD) and compared with the independent-sample t-test; skewed variables were presented as median [interquartile range (IQR)] and compared using the Mann–Whitney *U*-test. Categorical variables were summarized as counts (%) and analyzed using the *χ*^2^ test or Fisher’s exact test as appropriate. For regression modeling, continuous predictors such as gestational age, birth weight, PWR, and NLR were retained in their original continuous form to preserve information and interpretability.

### Statistical analysis

All analyses were performed using R software (version 4.3.3). Univariate logistic regression was first used to screen potential maternal and neonatal risk factors for BPD. Variables with *p* < 0.10 were entered into a multivariable logistic regression model to identify independent predictors and control for confounding. Adjusted odds ratios (aORs) and 95% confidence intervals (CIs) were reported. Model discrimination was evaluated by the area under the receiver operating characteristic curve (AUC) with 95% CI. Calibration was assessed using the Hosmer–Lemeshow goodness-of-fit test, bootstrap (1,000 resamples) correction, and calibration plots. Overall model fit was examined with the likelihood ratio test, and a nomogram was constructed based on the final logistic model coefficients. The optimal probability threshold was determined by the Youden index from ROC analysis. Statistical significance was set at *p* < 0.05 (two-tailed).

## Results

### Baseline characteristics of the study population

A total of 345 mother–infant pairs were included in this study, of whom 117 (33.9%) preterm infants developed bronchopulmonary dysplasia (BPD) and 228 (66.1%) did not. The baseline clinical and hematologic characteristics of mothers and neonates were summarized in Table 1. Among maternal variables, the mean maternal age was 30.4 ± 4.9 years, and the median gravidity was two (IQR 1–3), with no significant differences between the BPD and non-BPD groups (*p* > 0.05). However, the incidence of preeclampsia (25.6% vs. 16.2%, *p* = 0.04), premature rupture of membranes (PROM; 47.9% vs. 36.0%, *p* = 0.03), and chorioamnionitis (26.5% vs. 12.3%, *p* = 0.002) was significantly higher among mothers of infants who developed BPD. No significant group differences were observed in gestational diabetes mellitus, antenatal corticosteroid exposure, or cesarean delivery rate. Regarding maternal hematologic indices obtained within 24 h before delivery, mothers of infants with BPD showed higher white blood cell counts (median 10.1 [8.5–12.1] × 10^9^/L vs. 9.3 [7.7–10.8] × 10^9^/L, *p* = 0.01) and lower platelet counts (192 ± 54 vs. 215 ± 52 × 10^9^/L, *p* < 0.001). Consequently, the maternal platelet-to-white blood cell ratio (PWR) was significantly reduced in the BPD group (median 18.9 [IQR 15.2–23.5]) compared with the non-BPD group (23.1 [18.6–28.4], *p* < 0.001). In contrast, both the neutrophil-to-lymphocyte ratio (NLR; 5.9 vs. 4.7, *p* = 0.001) and platelet-to-lymphocyte ratio (PLR; 136 vs. 122, *p* = 0.02) were significantly elevated in mothers whose infants developed BPD. Neonatal characteristics also differed substantially between groups. Infants who developed BPD had a significantly lower mean gestational age (29.7 ± 2.4 weeks vs. 31.5 ± 2.1 weeks, *p* < 0.001) and birth weight (1,221 ± 312 g vs. 1,469 ± 295 g, *p* < 0.001). They were more likely to be male (61.5% vs. 48.7%, *p* = 0.03), small for gestational age (17.1% vs. 9.2%, *p* = 0.04), and to have lower 5-min Apgar scores (median 7 [IQR 6–8] vs. 8 (8, 9), *p* < 0.001). The incidences of both early-onset sepsis (16.2% vs. 5.7%, *p* = 0.002) and late-onset sepsis (29.1% vs. 10.1%, *p* < 0.001) were markedly higher in the BPD group. Moreover, prolonged mechanical ventilation (mechanical ventilation > 7 days) occurred in 54.7% of infants with BPD compared with only 10.5% of those without BPD (*p* < 0.001). As expected, all infants diagnosed with BPD required oxygen therapy for more than 28 days. Overall, mothers of infants who developed BPD exhibited a pro-inflammatory hematologic profile characterized by elevated white blood cell counts, decreased platelet counts, and lower PWR levels, while the affected neonates were born at an earlier gestational age and demonstrated more frequent infectious and respiratory complications (Table 1).

**Table 1:** Baseline characteristics of mothers and preterm infants according to bronchopulmonary dysplasia (BPD) status (*n* = 345).

Variable	Total (*n* = 345)	No BPD (*n* = 228)	BPD (*n* = 117)	*p*-value
Maternal characteristics				
Maternal age, y, mean ± SD	30.4 ± 4.9	30.2 ± 4.8	30.8 ± 5.0	0.42
Gravidity, median (IQR)	2 (1–3)	2 (1–3)	2 (1–3)	0.88
Preeclampsia, *n* (%)	67 (19.4)	37 (16.2)	30 (25.6)	0.04
Gestational diabetes mellitus (GDM), *n* (%)	42 (12.2)	31 (13.6)	11 (9.4)	0.28
Premature rupture of membranes (PROM), *n* (%)	138 (40.0)	82 (36.0)	56 (47.9)	0.03
Chorioamnionitis (clinical or histologic), *n* (%)	59 (17.1)	28 (12.3)	31 (26.5)	0.002
Antenatal corticosteroid exposure, *n* (%)	249 (72.2)	171 (75.0)	78 (66.7)	0.09
Cesarean delivery, *n* (%)	221 (64.1)	140 (61.4)	81 (69.2)	0.17
Maternal hematologic indices (within 24 h before delivery)				
White blood cell count (×10^9^/L), median (IQR)	9.6 (7.8–11.3)	9.3 (7.7–10.8)	10.1 (8.5–12.1)	0.01
Platelet count (×10^9^/L), mean ± SD	207 ± 53	215 ± 52	192 ± 54	<0.001
Platelet-to-white blood cell ratio (PWR), median (IQR)	21.8 (17.4–26.9)	23.1 (18.6–28.4)	18.9 (15.2–23.5)	<0.001
Neutrophil-to-lymphocyte ratio (NLR), median (IQR)	5.1 (3.7–6.8)	4.7 (3.4–6.2)	5.9 (4.3–7.9)	0.001
Platelet-to-lymphocyte ratio (PLR), median (IQR)	127 (101–155)	122 (98–149)	136 (108–165)	0.02
Neonatal characteristics				
Gestational age, weeks, mean ± SD	30.9 ± 2.3	31.5 ± 2.1	29.7 ± 2.4	<0.001
Birth weight, g, mean ± SD	1,384 ± 314	1,469 ± 295	1,221 ± 312	<0.001
Male sex, *n* (%)	183 (53.0)	111 (48.7)	72 (61.5)	0.03
Apgar score (5 min), median (IQR)	8 (7–9)	8 (8–9)	7 (6–8)	<0.001
Small for gestational age (SGA), *n* (%)	41 (11.9)	21 (9.2)	20 (17.1)	0.04
Early-onset sepsis, *n* (%)	32 (9.3)	13 (5.7)	19 (16.2)	0.002
Late-onset sepsis, *n* (%)	57 (16.5)	23 (10.1)	34 (29.1)	<0.001
Mechanical ventilation > 7 days, *n* (%)	88 (25.5)	24 (10.5)	64 (54.7)	<0.001
Oxygen therapy > 28 days, *n* (%)	117 (33.9)	0 (0)	117 (100)	—

Data are presented as mean ± standard deviation (SD), median [interquartile range (IQR)], or number (percentage). Continuous variables were compared using the independent-sample *t*-test or Mann–Whitney *U*-test as appropriate, and categorical variables were compared using the *χ*^2^ test or Fisher’s exact test. Percentages were calculated within each column; therefore, they do not sum to 100% across groups. PWR, platelet-to-white blood cell ratio (platelet count ÷ white blood cell count, both expressed in ×10^9^/L); NLR, neutrophil-to-lymphocyte ratio; PLR, platelet-to-lymphocyte ratio; PROM, premature rupture of membranes; GDM, gestational diabetes mellitus; SGA, small for gestational age. BPD was defined according to the 2018 NICHD consensus as oxygen dependency at 36 weeks’ postmenstrual age. All maternal hematologic indices were obtained within 24 h before delivery. *p* < 0.05 was considered statistically significant.

### Univariate logistic regression analysis of risk factors for bronchopulmonary dysplasia

Univariate logistic regression was performed to identify potential maternal and neonatal risk factors associated with bronchopulmonary dysplasia (BPD), and the results are summarized in Table 2. Among maternal characteristics, several obstetric complications were significantly associated with an increased risk of BPD. Preeclampsia increased the odds of BPD by approximately 1.8-fold (odds ratio [OR] = 1.78, 95% confidence interval [CI]: 1.01–3.13, *p* = 0.045), and premature rupture of membranes (PROM) was also positively related to BPD (OR = 1.60, 95% CI: 1.03–2.49, *p* = 0.037). The strongest maternal factor was clinical or histologic chorioamnionitis, which more than doubled the odds of BPD (OR = 2.56, 95% CI: 1.46–4.49, *p* = 0.001). Gestational diabetes mellitus, antenatal corticosteroid exposure, cesarean delivery, maternal age, and gravidity were not significantly associated with BPD occurrence (*p* > 0.05 for all). With respect to maternal hematologic indices measured within 24 h before delivery, elevated white blood cell count was positively associated with BPD (OR = 1.15 per ×10^9^/L increase, 95% CI: 1.04–1.27, *p* = 0.006), whereas higher platelet count was inversely associated (OR = 0.92 per 10 × 10^9^/L increase, 95% CI: 0.88–0.96, *p* < 0.001). Notably, the maternal platelet-to-white blood cell ratio (PWR) showed a robust negative association with BPD (OR = 0.93 per unit increase, 95% CI: 0.90–0.96, *p* < 0.001), indicating that mothers with lower PWR levels were more likely to deliver infants who subsequently developed BPD. In contrast, inflammatory ratios such as the neutrophil-to-lymphocyte ratio (NLR; OR = 1.18, 95% CI: 1.07–1.31, *p* = 0.001) and platelet-to-lymphocyte ratio (PLR; OR = 1.06 per 10-unit increase, 95% CI: 1.01–1.11, *p* = 0.019) were positively correlated with BPD risk, supporting an underlying pro-inflammatory maternal milieu. Regarding neonatal characteristics, lower gestational age and birth weight were the strongest independent correlates of BPD. Each additional gestational week reduced the odds of BPD by 37% (OR = 0.63, 95% CI: 0.55–0.72, *p* < 0.001), and each 100-g increase in birth weight decreased the odds by 21% (OR = 0.79, 95% CI: 0.72–0.86, *p* < 0.001). Male infants were at higher risk compared with female infants (OR = 1.68, 95% CI: 1.06–2.67, *p* = 0.027). Similarly, low 5-min Apgar scores (OR = 0.65 per point increase, 95% CI: 0.54–0.77, *p* < 0.001) and small-for-gestational-age (SGA) status (OR = 2.03, 95% CI: 1.02–4.06, *p* = 0.043) were associated with higher BPD incidence. Infectious complications were particularly influential: early-onset sepsis (OR = 3.17, 95% CI: 1.48–6.80, *p* = 0.003) and late-onset sepsis (OR = 3.71, 95% CI: 2.06–6.68, *p* < 0.001) were both significantly linked to BPD development. Prolonged mechanical ventilation (>7 days) demonstrated the highest odds among all predictors (OR = 9.68, 95% CI: 5.39–17.38, *p* < 0.001), underscoring the role of postnatal respiratory support in BPD pathogenesis. In summary, univariate analyses identified multiple prenatal and neonatal factors associated with BPD, including maternal inflammation (low PWR and high NLR/PLR), intrauterine infection, and early indicators of neonatal immaturity and respiratory compromise (Table 2).

**Table 2:** Univariate logistic regression analysis of risk factors associated with bronchopulmonary dysplasia (BPD) (*n* = 345).

Variable	OR	95% CI	*p*-value
Maternal characteristics			
Maternal age (per year)	1.02	0.97–1.07	0.42
Gravidity (≥3 vs. <3)	1.05	0.68–1.61	0.84
Preeclampsia (yes vs. no)	1.78	1.01–3.13	0.045
Gestational diabetes mellitus (GDM) (yes vs. no)	0.66	0.31–1.39	0.28
Premature rupture of membranes (PROM) (yes vs. no)	1.60	1.03–2.49	0.037
Chorioamnionitis (yes vs. no)	2.56	1.46–4.49	0.001
Antenatal corticosteroid exposure (yes vs. no)	0.66	0.41–1.06	0.09
Cesarean delivery (yes vs. no)	1.40	0.87–2.27	0.17
Maternal hematologic indices (within 24 h before delivery)			
White blood cell count (per ×10^9^/L increase)	1.15	1.04–1.27	0.006
Platelet count (per 10 × 10^9^/L increase)	0.92	0.88–0.96	<0.001
Platelet-to-white blood cell ratio (PWR) (per unit increase)	0.93	0.90–0.96	<0.001
Neutrophil-to-lymphocyte ratio (NLR) (per unit increase)	1.18	1.07–1.31	0.001
Platelet-to-lymphocyte ratio (PLR) (per 10 unit increase)	1.06	1.01–1.11	0.019
Neonatal characteristics			
Gestational age (per week increase)	0.63	0.55–0.72	<0.001
Birth weight (per 100 g increase)	0.79	0.72–0.86	<0.001
Male sex (yes vs. no)	1.68	1.06–2.67	0.027
Apgar score (5 min, per point increase)	0.65	0.54–0.77	<0.001
Small for gestational age (SGA) (yes vs. no)	2.03	1.02–4.06	0.043
Early-onset sepsis (yes vs. no)	3.17	1.48–6.80	0.003
Late-onset sepsis (yes vs. no)	3.71	2.06–6.68	<0.001
Mechanical ventilation >7 days (yes vs. no)	9.68	5.39–17.38	<0.001

Odds ratios (ORs) and 95% confidence intervals (CIs) were calculated from univariate logistic regression models with BPD as the dependent variable. Continuous predictors were analyzed per unit increase as indicated (parentheses specify the unit of change). PWR, platelet-to-white blood cell ratio (platelet count ÷ white blood cell count, both expressed in ×10^9^/L); NLR, neutrophil-to-lymphocyte ratio; PLR, platelet-to-lymphocyte ratio; PROM, premature rupture of membranes; GDM, gestational diabetes mellitus; SGA, small for gestational age. All maternal hematologic indices were measured within 24 h before delivery. *p* < 0.05 was considered statistically significant.

### Multivariable logistic regression analysis and identification of independent risk factors for BPD

Variables that showed significance or near-significance (*p* < 0.10) in univariate analyses were entered into a multivariable logistic regression model to identify independent predictors of bronchopulmonary dysplasia (BPD). The results are presented in Table 3.

**Table 3:** Multivariable logistic regression analysis of independent risk factors for bronchopulmonary dysplasia (BPD) (*n* = 345).

Variable	Adjusted OR	95% CI	*p*-value
Maternal variables			
Chorioamnionitis (yes vs. no)	1.94	1.01–3.71	0.046
Premature rupture of membranes (PROM) (yes vs. no)	1.52	0.90–2.56	0.11
Platelet-to-white blood cell ratio (PWR, per unit increase)	0.94	0.91–0.98	0.002
Neutrophil-to-lymphocyte ratio (NLR, per unit increase)	1.11	1.00–1.24	0.049
Neonatal variables			
Gestational age (per week increase)	0.68	0.58–0.79	<0.001
Birth weight (per 100 g increase)	0.86	0.78–0.94	0.001
Male sex (yes vs. no)	1.61	1.00–2.59	0.049
Early-onset sepsis (yes vs. no)	2.45	1.04–5.77	0.040
Late-onset sepsis (yes vs. no)	2.86	1.47–5.56	0.002
Mechanical ventilation > 7 days (yes vs. no)	6.74	3.34–13.62	<0.001

Adjusted odds ratios (ORs) and 95% confidence intervals (CIs) were estimated from a multivariable logistic regression model including variables with *p* < 0.10 in univariate analysis. PWR, platelet-to-white blood cell ratio (platelet count ÷ white blood cell count, both expressed in ×10^9^/L); NLR, neutrophil-to-lymphocyte ratio; PROM, premature rupture of membranes. All maternal hematologic indices were measured within 24 h before delivery. *p* < 0.05 was considered statistically significant.

After adjustment for potential confounders, several maternal and neonatal factors remained independently associated with the development of BPD. Among maternal variables, clinical or histologic chorioamnionitis was independently related to BPD (adjusted odds ratio [aOR] = 1.94, 95% confidence interval [CI]: 1.01–3.71, *p* = 0.046), indicating that intrauterine infection substantially increased the risk of postnatal pulmonary injury. In contrast, premature rupture of membranes (PROM) showed only a non-significant trend (aOR = 1.52, 95% CI: 0.90–2.56, *p* = 0.11). Notably, the maternal platelet-to-white blood cell ratio (PWR) remained significantly associated with BPD, with a lower PWR associated with higher BPD risk (aOR = 0.94 per unit increase, 95% CI: 0.91–0.98, *p* = 0.002). Conversely, a higher maternal neutrophil-to-lymphocyte ratio (NLR) was modestly but significantly related to increased BPD odds (aOR = 1.11, 95% CI: 1.00–1.24, *p* = 0.049), supporting the hypothesis that a systemic maternal inflammatory state contributes to disease susceptibility. Among neonatal variables, gestational age and birth weight remained the strongest protective factors. Each additional gestational week reduced the odds of BPD by approximately 32% (aOR = 0.68, 95% CI: 0.58–0.79, *p* < 0.001), and each 100-g increase in birth weight was associated with a 14% reduction in BPD risk (aOR = 0.86, 95% CI: 0.78–0.94, *p* = 0.001). Male sex was independently associated with higher BPD risk (aOR = 1.61, 95% CI: 1.00–2.59, *p* = 0.049). Neonatal infectious morbidities also remained significant predictors: early-onset sepsis (aOR = 2.45, 95% CI: 1.04–5.77, *p* = 0.040) and late-onset sepsis (aOR = 2.86, 95% CI: 1.47–5.56, *p* = 0.002) were both associated with approximately two- to three-fold increased odds of developing BPD. Mechanical ventilation for more than 7 days was the most powerful independent predictor, with a nearly seven-fold higher risk of BPD (aOR = 6.74, 95% CI: 3.34–13.62, *p* < 0.001). Collectively, the final multivariable model identified maternal inflammation (low PWR, high NLR, and chorioamnionitis) and markers of neonatal immaturity and respiratory morbidity (lower gestational age, low birth weight, prolonged ventilation, and neonatal sepsis) as the major independent contributors to BPD development (Table 3).

### Model performance and predictive ability

**Figure 1:**
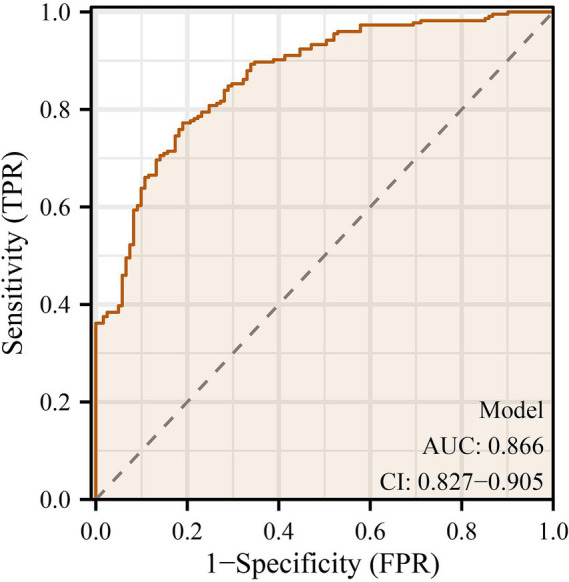
Receiver operating characteristic (ROC) curve of the multivariable prediction model for bronchopulmonary dysplasia (BPD). The receiver operating characteristic (ROC) curve illustrates the discriminative performance of the final multivariable logistic regression model for predicting bronchopulmonary dysplasia (BPD) in preterm infants. The area under the curve (AUC) was 0.866 (95% confidence interval [CI]: 0.827–0.905), indicating excellent discrimination between infants who developed BPD and those who did not. The diagonal dashed line represents the reference line of no discrimination (AUC = 0.5). The model demonstrated high sensitivity at low false-positive rates, reflecting strong predictive ability based on maternal platelet-to-white blood cell ratio (PWR) and neonatal clinical parameters.

**Figure 2:**
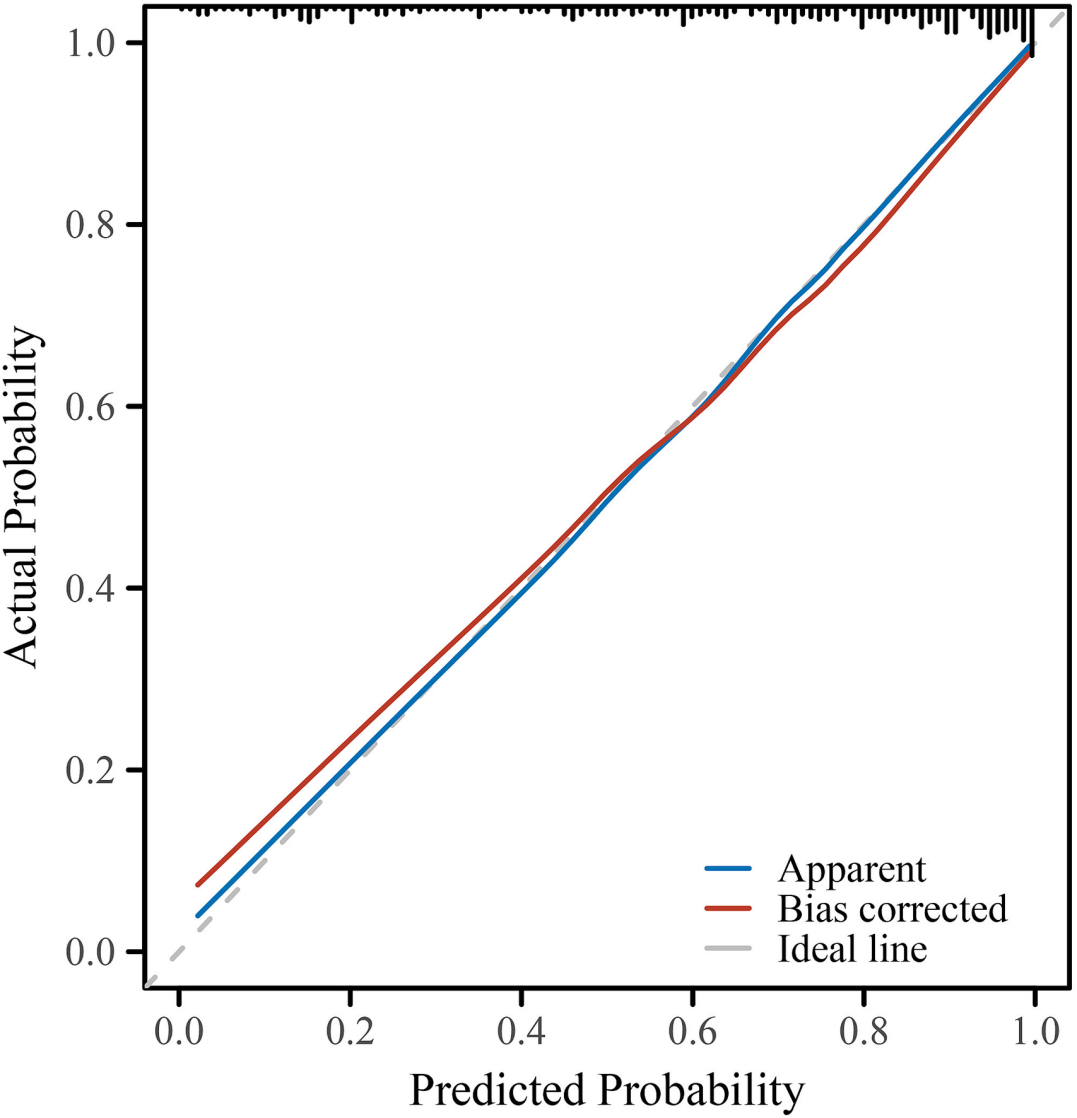
Calibration plot of the predictive model for bronchopulmonary dysplasia (BPD). Calibration performance of the final multivariable logistic regression model for predicting bronchopulmonary dysplasia (BPD) in preterm infants. The blue line represents the apparent calibration in the original dataset, while the red line indicates the bias-corrected calibration curve obtained after 1,000 bootstrap resamples. The dashed gray line represents the ideal reference line (perfect agreement between predicted and observed probabilities). The close alignment of the apparent and bias-corrected lines with the ideal line indicates excellent model calibration, suggesting that the predicted probabilities of BPD closely matched the actual observed outcomes.

**Figure 3:**
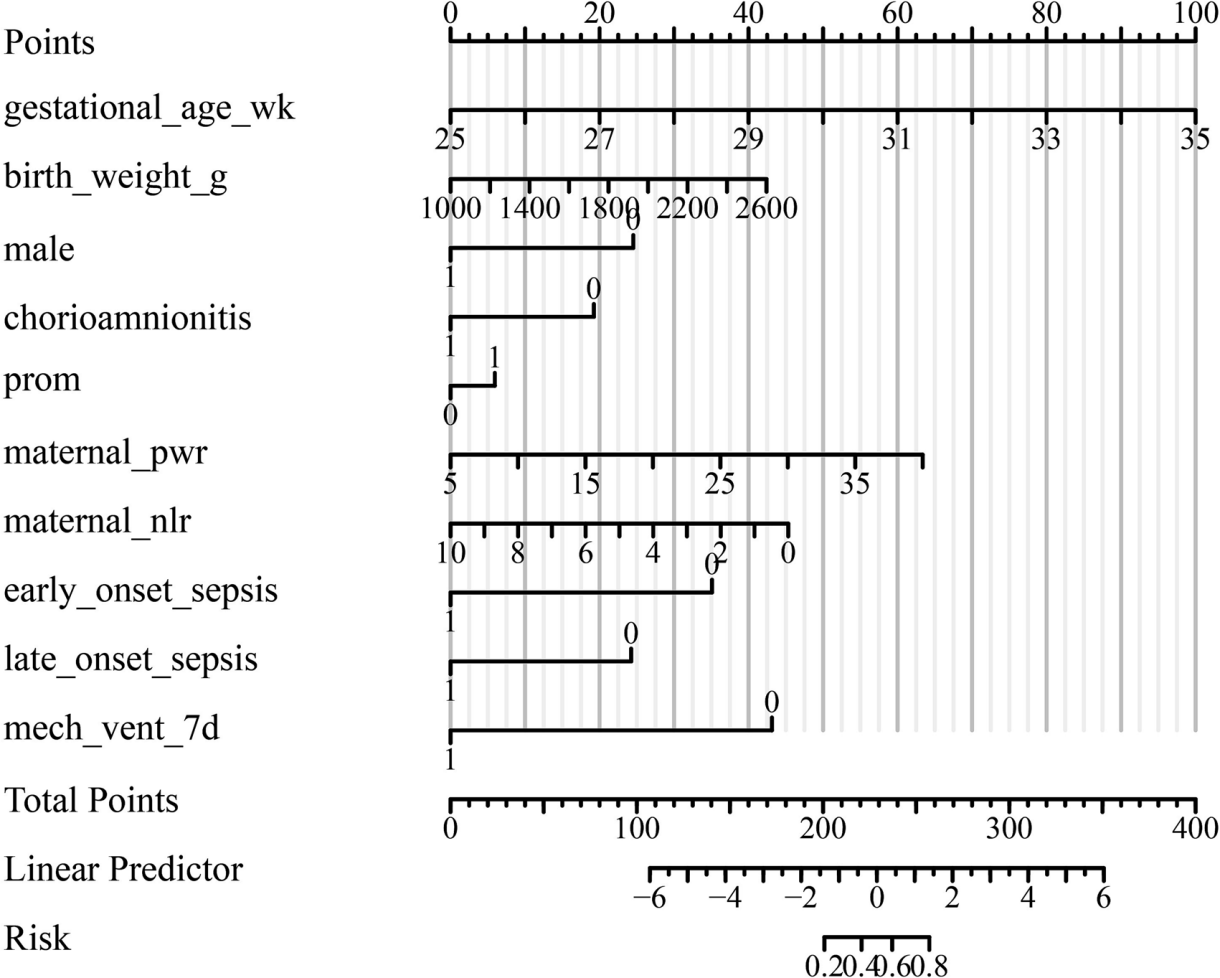
Nomogram for predicting the risk of bronchopulmonary dysplasia (BPD) in preterm infants. The nomogram was developed based on the final multivariable logistic regression model to estimate the individual probability of bronchopulmonary dysplasia (BPD) among preterm infants. Each predictor variable [maternal platelet-to-white blood cell ratio (PWR), gestational age, birth weight, neonatal sex, chorioamnionitis, premature rupture of membranes (PROM), early-onset sepsis, late-onset sepsis, and mechanical ventilation >7 days] is assigned a corresponding score on the “Points” scale. The total score, obtained by summing the individual points for all predictors, corresponds to a predicted probability of BPD at the bottom of the plot. Higher total points indicate an increased risk of developing BPD. The nomogram provides a visual and practical tool for early individualized risk assessment in clinical settings.

The multivariable logistic regression model incorporating maternal platelet-to-white blood cell ratio (PWR) and key neonatal clinical variables demonstrated excellent performance for predicting bronchopulmonary dysplasia (BPD) (Table 4). The area under the ROC curve (AUC) was 0.866 (95% CI: 0.827–0.905), indicating strong discrimination between infants who developed BPD and those who did not (Figure 1). At the optimal cut-off probability of 0.763, the model achieved a sensitivity of 77.2%, specificity of 80.9%, and overall accuracy of 78.6%, with positive and negative predictive values of 88.3 and 65.8%, respectively. Model calibration was excellent, as shown by the Hosmer–Lemeshow goodness-of-fit test (*χ*^2^ = 10.833, *p* = 0.211) and the bootstrap-corrected calibration slope of 0.98, both indicating good agreement between predicted and observed probabilities (Figure 2). The likelihood ratio test confirmed that the model as a whole was statistically significant (*χ*^2^ = 152.2, *p* < 0.001). A nomogram was subsequently constructed to facilitate clinical application (Figure 3). Higher total points corresponded to increased predicted risk of BPD, with gestational age, birth weight, prolonged mechanical ventilation, and low maternal PWR contributing most substantially to risk estimation. These findings collectively demonstrate that the model achieved high discrimination, good calibration, and strong clinical interpretability, supporting its potential use as an individualized prediction tool for early identification of high-risk preterm infants. For ease of reference, a concise summary of all independent predictors included in the final multivariable model is provided in Supplementary Table 1.

**Table 4:** Performance of the multivariable prediction model for bronchopulmonary dysplasia (BPD).

Evaluation category	Statistic	Estimate (95% CI or value)	*p*-value/Interpretation
Discrimination	Area under the ROC curve (AUC)	0.866 (0.827–0.905)	–
C-index	0.866 (0.827–0.905)	*p* < 0.001	
Calibration	Hosmer–Lemeshow goodness-of-fit test	*χ*^2^ = 10.833	*p* = 0.211 → Good fit
Calibration slope (bootstrap-corrected)	0.98	–	
Overall model fit	Likelihood ratio test	*χ*^2^ = 152.2	*p* < 0.001
Optimal cut-off (Youden index)	Probability threshold	0.763	–
Sensitivity	0.772		
Specificity	0.810		
Accuracy	0.786		
Youden index	0.582		
Predictive values at optimal cut-off	Positive predictive value (PPV)	0.883	–
Negative predictive value (NPV)	0.658	–	

Model performance was assessed in the derivation cohort (*n* = 345) using internal validation with 1,000 bootstrap resamples. Discrimination was evaluated by the area under the receiver operating characteristic curve (AUC = 0.866, 95% CI: 0.827–0.905), indicating excellent ability to distinguish infants who developed bronchopulmonary dysplasia (BPD) from those who did not. Calibration was assessed by the Hosmer–Lemeshow test (*p* = 0.211), demonstrating good agreement between predicted and observed probabilities, and visually confirmed by the calibration plot (Figure 2). The model achieved optimal sensitivity (0.77) and specificity (0.81) at a cut-off probability of 0.763, with a Youden index of 0.582. The overall likelihood ratio test was highly significant (*p* < 0.001), confirming that the model provided a statistically meaningful improvement in prediction. BPD, bronchopulmonary dysplasia; AUC, area under the curve; CI, confidence interval; PPV, positive predictive value; NPV, negative predictive value. *p* < 0.05 was considered statistically significant.

## Discussion

In this retrospective cohort of 345 mother–infant pairs, we found that a lower maternal platelet-to-white blood cell ratio (PWR) measured within 24 h before delivery was independently associated with the development of bronchopulmonary dysplasia (BPD) in preterm infants. Even after adjustment for established neonatal predictors, including gestational age, birth weight, sepsis, and prolonged mechanical ventilation (mechanical ventilation > 7 days), PWR remained significantly associated with the development of BPD in the multivariable model. The model demonstrated excellent discrimination (AUC = 0.866) and calibration, suggesting that maternal inflammatory status, reflected by hematologic indices, provides additive value in early risk stratification for BPD. These findings highlight the maternal inflammatory contribution to neonatal pulmonary morbidity and underscore the utility of a simple, readily available biomarker in prediction modeling.

Our findings align with accumulating evidence that maternal inflammation and intrauterine immune activation are critical determinants of BPD pathogenesis (11, 15). In our cohort, a lower maternal PWR and a higher NLR within 24 h before delivery, together with clinical or histologic chorioamnionitis, were all independently associated with subsequent BPD. Taken together, these markers support the concept of a pro-inflammatory maternal milieu that predisposes the fetal lung to injury. Prior work has predominantly focused on neonatal inflammatory biomarkers—such as C-reactive protein, IL-6, or early postnatal NLR—as predictors of BPD risk (16, 17), whereas maternal hematologic indices have received far less attention. The strong inverse association between PWR and BPD in our study suggests that relative thrombocytopenia in the context of leukocytosis may capture an exaggerated systemic inflammatory response at the maternal–fetal interface. Consistent with this interpretation, Jain et al. (10) showed that histologic chorioamnionitis increases the risk of BPD, and Ramos-Navarro et al. (9) reported that placental inflammatory lesions are closely linked to BPD, with gestational age and sex further modifying this association. By integrating PWR, NLR, and chorioamnionitis into a single model, our results extend these observations and highlight maternal hematologic ratios as practical, non-invasive surrogates of peripartum inflammatory burden that may help identify high-risk dyads before or at the time of delivery.

In addition to maternal factors, our multivariable model confirmed and refined the role of several well-established neonatal predictors of BPD. As expected, lower gestational age and birth weight remained the most powerful protective factors, with each additional week of gestation and each 100-g increase in birth weight substantially reducing the odds of BPD. These findings are consistent with prior epidemiologic and prediction studies showing that extreme prematurity and very low birth weight are the primary drivers of BPD risk (18, 19). We also observed that male sex independently increased the likelihood of BPD, which echoes reports that male infants exhibit greater vulnerability to respiratory morbidity and a higher incidence of BPD than females, possibly due to sex-related differences in lung maturation and hormonal regulation (9, 19). Furthermore, early-onset sepsis (EOS) and late-onset sepsis (LOS) were both associated with approximately two- to three-fold higher odds of BPD, underscoring the contribution of systemic infection and sustained inflammatory exposure to ongoing lung injury (11, 16). Finally, prolonged mechanical ventilation (>7 days) emerged as the strongest predictor in our model, consistent with the established link between invasive ventilation, ventilator-induced lung injury, and the development of “new BPD” characterized by impaired alveolar and vascular growth (11, 18). Clinically, these results emphasize that BPD arises from the convergence of antenatal inflammatory priming and postnatal hazards such as infection and prolonged ventilatory support.

In contrast to prior models relying solely on neonatal variables—such as gestational age, birth weight, or respiratory support requirements (18, 19)—our model deliberately integrates maternal hematologic indices with key neonatal characteristics. By jointly considering maternal PWR, NLR, chorioamnionitis, gestational age, birth weight, sex, sepsis, and duration of mechanical ventilation, the model captures both prenatal susceptibility and postnatal disease expression. This study, therefore, provides novel evidence linking low maternal PWR to BPD, while at the same time confirming the independent contributions of prematurity, infection, and ventilator exposure. Our findings extend previous research on NLR and PLR as predictors of perinatal inflammatory complications such as preeclampsia and preterm labor (12, 13, 17), and suggest that maternal hematologic ratios can be meaningfully incorporated into BPD risk stratification frameworks.

The biological plausibility of PWR as a predictor of BPD is supported by its ability to capture systemic inflammatory activity. Platelets participate in inflammation through cytokine release and endothelial interactions, whereas leukocytosis indicates immune activation and tissue stress (20). A lower PWR, therefore, implies platelet consumption and leukocyte proliferation—hallmarks of systemic inflammation. During pregnancy, such maternal inflammatory activation can alter the placental–fetal interface, inducing cytokine cascades and oxidative stress that impair pulmonary angiogenesis and alveolarization (21). Experimental studies have shown that intrauterine exposure to lipopolysaccharide or elevated maternal IL-6 triggers fetal inflammatory response syndrome, resulting in arrested lung development and heightened susceptibility to oxygen- or ventilation-induced injury after birth (15). From a clinical standpoint, a low maternal PWR in the peripartum period may therefore serve as a readily obtainable warning signal of an exaggerated inflammatory state that warrants heightened surveillance and anticipatory respiratory care for the preterm infant.

Furthermore, inflammation-induced platelet dysfunction may compromise vascular integrity and microcirculatory homeostasis, both central to “new BPD,” which is characterized by disrupted pulmonary vascular and alveolar growth rather than fibroproliferative scarring (22). The observed association between low maternal PWR and neonatal BPD in our study, therefore, likely reflects an underlying inflammatory–vascular mechanism that begins *in utero* and continues postnatally under additional stressors such as infection and mechanical ventilation.

This study suggests that maternal PWR, an inexpensive and routinely available hematologic index, may serve as an early screening tool for identifying pregnancies at high risk for neonatal BPD. Incorporating maternal inflammatory markers into predictive frameworks could allow clinicians to recognize vulnerable dyads before or shortly after delivery, enabling tailored preventive strategies, such as optimized perinatal infection control, judicious ventilation practices, and closer respiratory follow-up. Because PWR can be derived from a standard complete blood count, its implementation would impose minimal cost or logistic burden, especially in resource-limited neonatal care settings.

The present findings have several potential implications for clinical practice. First, because maternal PWR and NLR are derived from a routine complete blood count, they offer an inexpensive and readily available approach to identify pregnancies in which the fetus may be exposed to a heightened inflammatory milieu. In settings where preterm delivery is anticipated, a low maternal PWR in the peripartum period could prompt closer antenatal and immediate postnatal surveillance of the infant’s respiratory status, particularly when combined with other antenatal risk factors such as chorioamnionitis. Second, by integrating maternal hematologic indices with key neonatal variables—including gestational age, birth weight, sex, sepsis, and duration of mechanical ventilation—the nomogram provides a pragmatic tool for individualized BPD risk estimation during the early NICU course. Clinicians may use this information to prioritize non-invasive respiratory support, minimize unnecessary invasive ventilation, optimize infection prevention strategies, and plan more intensive follow-up for infants at the highest risk.

From a nursing perspective, early identification of high-risk mother–infant dyads based on maternal PWR and the nomogram could support targeted allocation of nursing resources, such as enhanced respiratory monitoring, anticipatory parental education about BPD, and early engagement in multidisciplinary care pathways. Importantly, we do not propose that PWR or the model replace clinical judgment or established decision-making frameworks. Rather, they should be viewed as adjunctive tools that can complement existing clinical assessments. External and temporal validation in larger, multi-center cohorts will be essential before routine implementation, but our results provide a preliminary foundation for incorporating maternal inflammatory markers into standardized risk assessment and perinatal care protocols for preterm infants.

Several limitations should be acknowledged. First, this was a single-center retrospective cohort based on routinely collected electronic records; as such, selection bias and information bias are possible, and causal inference cannot be drawn. Second, maternal hematologic indices were obtained at a single pre-delivery time point and may be influenced by the timing of sampling relative to labor, membrane rupture, antibiotics, or corticosteroid administration; serial measurements could better characterize inflammatory dynamics. Third, PWR is an indirect, composite marker that cannot delineate specific cytokine pathways or platelet function; integrating placental pathology, maternal cytokines, or platelet activation indices would strengthen mechanistic interpretation. Fourth, although multivariable modeling adjusted for major confounders and internal validation (bootstrap) supported model stability, residual confounding from unmeasured factors—such as detailed antenatal medication dosing (e.g., corticosteroids, magnesium sulfate), maternal nutritional status, and standardized placental histology—cannot be excluded. Fifth, our predictive framework included postnatal variables such as prolonged mechanical ventilation; while this improves discrimination for clinical prediction, it may also capture mediators on the causal pathway from antenatal inflammation to BPD. Future work should therefore evaluate both an “early-prediction” model limited to pre/perinatal variables and a “full clinical” model for bedside risk stratification. Sixth, BPD classification followed NICHD 2018 criteria using charted oxygen/respiratory support, which may be susceptible to misclassification if device settings (e.g., high-flow nasal cannula flow/FiO₂) are inconsistently documented; standardized assessments at 36 weeks’ PMA would reduce this risk. Finally, despite internal bootstrap correction, the model was not externally or temporally validated; confirmation in multi-center cohorts with different laboratory platforms and care practices, along with evaluation across gestational-age strata and determination of clinically actionable PWR thresholds, is required to establish generalizability and guide implementation.

In summary, our study identifies a lower maternal platelet-to-white blood cell ratio before delivery as an independent and biologically plausible predictor of BPD in preterm infants. This finding reinforces the concept that the maternal inflammatory milieu exerts a lasting influence on neonatal pulmonary outcomes. From a nursing perspective, early recognition of maternal inflammatory risk through simple hematologic indices such as PWR may enable neonatal nurses and perinatal care teams to implement anticipatory interventions—such as closer respiratory monitoring, individualized oxygen therapy, and infection prevention strategies—in high-risk preterm infants. Moreover, integrating maternal biomarkers into multidisciplinary perinatal nursing protocols could strengthen continuity of care from the obstetric to neonatal period. Future prospective, multi-center studies should validate the prognostic accuracy of PWR, investigate its interaction with specific cytokine networks, and explore whether maternal anti-inflammatory or immunomodulatory interventions, alongside comprehensive nursing support, can mitigate BPD risk. Integrating maternal biomarkers such as PWR into predictive models represents a promising step toward precision perinatal medicine and mother–infant-integrated preventive nursing for BPD.

## Data Availability

The original contributions presented in the study are included in the article/Supplementary material, further inquiries can be directed to the corresponding author.
