# Supplementary material for: Maternal inflammatory biomarkers and neonatal characteristics as predictors of bronchopulmonary dysplasia in preterm infants: a retrospective cohort study
Source: Front Med (Lausanne). 2026 Jan 8;12:1735582. doi: 10.3389/fmed.2025.1735582 (PMC12823951; doi:10.3389/fmed.2025.1735582)
Supplement: Supplementary file 1 [file Table_1.docx]

**Supplementary Table 1.** Summary of key independent predictors of bronchopulmonary dysplasia (BPD) in the final multivariable model

| **Predictor** | **Direction of association** | **Adjusted OR (95% CI)** | **Clinical interpretation** |
| --- | --- | --- | --- |
| **Chorioamnionitis (yes vs no)** | Higher risk in exposed infants | 1.94 (1.01–3.71) | Intrauterine infection increases susceptibility to postnatal lung injury. |
| **Maternal PWR (per unit increase)** | Lower PWR → higher risk | 0.94 (0.91–0.98) | Low PWR reflects heightened maternal inflammatory burden. |
| **Maternal NLR (per unit increase)** | Higher NLR → higher risk | 1.11 (1.00–1.24) | Elevated NLR indicates systemic inflammation that may prime fetal lungs. |
| **Gestational age (per week increase)** | Lower GA → higher risk | 0.68 (0.58–0.79) | Extreme prematurity remains the principal determinant of BPD. |
| **Birth weight (per 100 g increase)** | Lower BW → higher risk | 0.86 (0.78–0.94) | Small infants are more vulnerable to lung injury. |
| **Male sex (yes vs no)** | Higher risk in males | 1.61 (1.00–2.59) | Male infants show delayed pulmonary maturation. |
| **Early-onset sepsis (EOS)** | Higher risk with EOS | 2.45 (1.04–5.77) | Early systemic infection accelerates inflammatory injury. |
| **Late-onset sepsis (LOS)** | Higher risk with LOS | 2.86 (1.47–5.56) | Recurrent inflammation contributes to cumulative lung injury. |
| **Mechanical ventilation > 7 days** | Markedly higher risk | 6.74 (3.34–13.62) | Prolonged invasive ventilation is the strongest postnatal predictor. |

**Note:**PWR = platelet-to-white blood cell ratio; NLR = neutrophil-to-lymphocyte ratio; EOS = early-onset sepsis; LOS = late-onset sepsis; OR = odds ratio; CI = confidence interval. All predictors shown were identified from the final multivariable logistic regression model. Higher OR values indicate predictors associated with increased risk of bronchopulmonary dysplasia.
